# *Synopeas ruficoxum* Buhl (Hymenoptera, Platygastridae) is a natural enemy of soybean gall midge, *Resseliella maxima* Gagné (Diptera, Cecidomyiidae)

**DOI:** 10.3897/jhr.98.163211

**Published:** 2025-08-25

**Authors:** Sarah C. von Gries, Jessica Awad, Elijah J. Talamas, Anthony J. McMechan, Robert L. Koch, Amelia R. I. Lindsey

**Affiliations:** 1 Department of Entomology, University of Minnesota, 1980 Folwell Ave, Saint Paul, MN 55108, USA; 2 Naturalis Biodiversity Center, Darwinweg 2, 2333 CR Leiden, Netherlands; 3 Florida Department of Agriculture and Consumer Services, Division of Plant Industry, 1911 SW 34th St, Gainesville FL 32608, USA; 4 Eastern Nebraska Research, Extension, and Education Center, University of Nebraska - Lincoln, 1071 Co Rd G, Ithaca, NE, 68033, USA

**Keywords:** biological control, host association, parasitism, *Synopeas maximum*, *Wolbachia*, parthenogenesis

## Abstract

Platygastridae (Hymenoptera) is known as a ‘dark taxon’ as it is highly diverse and understudied. Within Platygastridae, one of the largest genera is *Synopeas* Förster, species of which parasitize Cecidomyiidae (Diptera). This study identifies a new host association between these two families, with *Synopeas ruficoxum* Buhl as the second reported parasitoid of soybean gall midge, *Resseliella maxima* Gagné. Parasitoids were reared from soybean stems infested with *R. maxima* collected in Nebraska, USA. Furthermore, PCR assays confirmed that *R. maxima* larvae are parasitized by *S. ruficoxum* in the field. All *S. ruficoxum* specimens were female, suggesting that this may be an asexually reproducing population. We found that some, but not all, *S. ruficoxum* were infected with a bacterium, *Wolbachia*, known to mediate asexual reproduction in other insects, suggesting other factors may be responsible for the all-female population. Publicly available barcoding data allowed us to determine that *S. ruficoxum* is also present in Eastern Canada, which is beyond the known geographic range of *R. maxima*. This suggests that *S. ruficoxum* has other hosts or that the geographic range of *R. maxima* is broader than currently documented. A redescription and diagnostic data for *S. ruficoxum* are provided, advancing the ability to use this parasitoid for biological control of *R. maxima*.

## Introduction

Platygastridae (Hymenoptera) is known as a ‘dark taxon’, a term used to describe understudied taxa that are highly diverse, difficult to identify, and in need of professional taxonomic organization and identification (Srivathsan et al. 2022; Awad et al. 2023). Within Platygastridae, *Synopeas* Förster, 1856, is one of the largest genera, containing close to 400 described species (Awad et al. 2021). *Synopeas* species are koinobiont endoparasitoids, which means they oviposit into eggs or young larvae and the parasitoid waits to metamorphose until the last larval instar or prepupal stage of the host (Austin, 1984; Kim et al. 2011; Abram et al. 2012; Chen et al. 2021). *Synopeas* species are only known to parasitize Cecidomyiidae (Diptera), and many species appear to be host-specific, although relatively few host records exist (Vlug 1995; Awad et al. 2021).

Cecidomyiidae is also a diverse dark taxon, found globally, with new species regularly being discovered and described (Huang et al. 2022; Srivathsan et al. 2022). The soybean gall midge, *Resseliella maxima* Gagné, 2019 (Diptera: Cecidomyiidae) is one such recently described species (Gagné et al. 2019). *Resseliella maxima* has been reported in seven states in the midwestern USA (McMechan et al. 2023), where it is a pest of soybean, *Glycine max* (L.) Merr. (Fabalaes: Fabaceae), with potential to cause high yield reductions (McMechan et al. 2021; Helton et al. 2022). *Resseliella maxima* has also been found to infest other Fabaceae, including sweet clover (*Melilotus officinalis* (L.) Lam.), alfalfa (*Medicago sativa* L.), and dry beans (*Phaseolus vulgaris* L.) (Potter et al. 2022; Bragard et al. 2023). However, it remains unknown if *R. maxima* is a previously unknown exotic species that invaded the USA or if it is a native that expanded its host range to include soybean.

Recent surveys for natural enemies of *R. maxima* in Minnesota led to the discovery of *Synopeas maximum* Awad & Talamas, 2023 (Melotto et al. 2023a), which was confirmed to parasitize *R. maxima* (Melotto et al. 2023b). The work presented here documents a second species, *Synopeas ruficoxum* Buhl, 2006 that also parasitizes *R. maxima*. The original description of *S. ruficoxum* is brief, based solely on the morphology of a single female specimen (Buhl 2006), and is insufficient for diagnosis. Therefore, a morphological and molecular redescription of *S. ruficoxum* is provided. This study also marks the first record of *S. ruficoxum* from the United States.

## Methods

### Field collection and laboratory rearing

*Synopeas* specimens were acquired in 2021 and 2023 using methods modified from Melotto et al. (2023b). In brief, *R. maxima*-infested soybean stems were collected from soybean field edges, placed into emergence buckets in the laboratory, and monitored for emergence of adult insects. These adult insects were collected, freeze-killed, and preserved in 95% ethanol for morphological and molecular identification. In 2021, *R. maxima*-infested soybean stems were collected on 24 and 27 August from two fields in Lancaster County, Nebraska. Emerged adult insects from 2021 were then pooled together for preservation and storage. In 2023, *R. maxima*-infested soybean stems were collected from two fields in Nebraska, one field near the city of Syracuse (Otoe County) and the other near the city of Wahoo (Saunders County). Fields were sampled every two weeks starting when fields began to show signs of infestation (Syracuse: 22 June; Wahoo: 27 June) and continued until soybean plants senesced (Syracuse: 18 August; Wahoo: 25 August). Emerged adult insects from 2023 were then separated by field and sampling date for preservation and storage.

### DNA barcoding

Genomic DNA from individual specimens was extracted using a modified non-destructive HotSHOT protocol (Truett et al. 2000), as described in Melotto et al. (2023b). The cytochrome oxidase subunit I (COI) barcoding region was amplified with the universal primer pair LCO-1490/HCO-2198 (Folmer et al. 1994). Since all of the reared wasps of interest were female (see results), specimens were screened for *Wolbachia*, an endosymbiont found in insects and known to alter sex ratios (Werren et al. 2008). *Wolbachia*-specific primers Wspec_F/Wspec_R (Werren & Windsor, 2000) were used to amplify the 16S rRNA gene. All PCR reactions were prepared in a final volume of 20 μL with Q5 Hot Start High-Fidelity 2X Master Mix (New England BioLabs), 1 μL of DNA template, and 500 nM of each primer alongside positive and negative controls. Thermalcycling was performed on a Mastercycler nexus PCR cycler (Eppendorf ) with an initial denaturation of 2 min at 98 °C, followed by 35 cycles of amplification (COI: 10 s at 98 °C, 30 s at 55 °C, and 20 s at 72 °C; Wspec: 15 s at 98 °C, 15 s at 60 °C, and 15 s at 72 °C), and a final elongation of 2 min at 72 °C. PCR products were separated on a 1% agarose gel via electrophoresis and imaged under ultraviolet light after staining with 3X GelRed (Biotium). The COI PCR products were cleaned with the DNA Clean & Concentrator-5 Kit (Zymo Research) according to the manufacturer’s instructions and sequenced in both directions via Sanger sequencing (ACGT, Inc. Wheeling, IL, USA). Sequences were inspected for peak quality, aligned, and trimmed of priming regions in SnapGene version 6.2.1. The COI sequences were deposited in BOLD (Barcode of Life Data System) and accession numbers are listed in Table 1.

### Phylogenetic analysis

BLASTn was used to query COI barcodes from Nebraska specimens against GenBank and identify putative conspecifics. Then, a phylogenetic reconstruction of *Synopeas* was performed with all unique *S. ruficoxum* haplotypes, previously published *Synopeas* sequences available on BOLD, and an outgroup from the genus *Leptacis* Förster, 1856 (Hymenoptera: Platygastridae) (Table 2). Specific *Synopeas* sequences were selected based on the previously published *Synopeas* phylogeny from Melotto et al. (2023a) to ensure breadth across the genus and resolution within the subgroup to which *S. ruficoxum* belongs. Sequences were aligned using MAFFT version 7.475 (Katoh and Standley 2013) with default parameters and manually inspected to confirm that codons were aligned (*i.e.*, no frameshifts). Phylogenetic reconstruction was performed with IQ-Tree version 1.6.12 using model optimization option (TIM+F+I+G4 model selected) and 1000 ultrafast bootstrap replicates. The tree was rooted and formatted in FigTree version 1.4.4 and annotated in Inkscape version 1.3.2.

### Parasitoid-host association

To test the parasitoid-host association between *S. ruficoxum* and *R. maxima*, field-collected *R. maxima* larvae were screened for parasitism using PCR. Larvae were obtained by collecting *R. maxima*-infested soybean stems and dissecting out the larvae, as per Melotto et al. (2023b). Two stems were collected from the edge of the aforementioned field in Wahoo on 27 June and 11 July 2023. Of the larvae dissected from the stems, seven were randomly selected to be screened for parasitism by *S. ruficoxum*. DNA from individual larvae was extracted using a modified destructive protocol (Truett et al. 2000), as described in Melotto et al. (2023b).

For the detection and identification of *S. ruficoxum* DNA in *R. maxima* larvae, a *S. ruficoxum*-specific reverse primer (Sruf_Rmax_R: GATTCTAATATCAATTGAAGC) was designed. This primer was paired with the universal primer LCO-1490 (Folmer et al. 1994) to amplify a 370 bp region of COI. PCR reactions were prepared as above and thermal cycling was performed with an initial denaturation of 2 min at 98 °C, followed by 35 cycles of amplification (10 s at 98 °C, 30 s at 54 °C, and 20 s at 72 °C), and a final elongation of 2 min at 72 °C. Controls included a no-template negative control and DNA extracted from an *S. ruficoxum* adult as a positive control. The PCR products were separated, cleaned, and sequenced in the same manner as described above to test if the PCR amplicon was in fact derived from *S. ruficoxum*.

In parallel, to verify these larvae were *R. maxima* and not a different cecidomyiid, the larvae were barcoded using the universal degenerate primer pair LCO-1490-JJ2/HCO2198-JJ2 (Astrin et al. 2016), then aligned to a confirmed *R. maxima* sequence (GenBank accession number OQ342780). PCR reactions were prepared as above and thermal cycling was performed with an initial denaturation of 2 min at 98 °C, followed by 35 cycles of amplification (10 s at 98 °C, 30 s at 55 °C, and 20 s at 72 °C), and a final elongation of 2 min at 72 °C. The PCR products were separated, cleaned, and sequenced in the same manner as mentioned above.

### Imaging

Brightfield photography was performed using a Macropod microphotography system (Macroscopic Solutions) with 10× and 20× Mitutoyo objective lenses. Scanning electron microscopy was performed with a Phenom XL G2 Desktop SEM. Image stacks were rendered in Helicon Focus, and images of primary types were deposited in Zenodo (Table 3). Images of reared voucher specimens were deposited in BOLD (Table 1).

### Institutional abbreviations

Specimens examined during this study are deposited in the following institutions and abbreviated as follows:

**Table:** 

**HNHM**	Hungarian Museum of Natural History, Budapest, Hungary
**KUEC**	Kyushu University Entomological Collection, Fukuoka, Japan
**MNCN**	Museo Nacional de Ciencias Naturales, Madrid, Spain
**MZLU**	Lund University Zoological Museum, Lund, Sweden
**NBC**	Naturalis Biodiversity Center, Leiden, Netherlands
**NHMD**	Natural History Museum of Denmark, Copenhagen, Denmark
**NHMUK**	Natural History Museum, London, UK
**NMINH**	National Museum of Ireland, Natural History, Dublin, Ireland
**RMCA**	Royal Museum for Central Africa, Tervuren, Belgium
**USNM**	United States National Museum, Washington DC, USA

## Results

### Laboratory rearing

From the *R. maxima*-infested stems collected in Nebraska in 2021 and 2023, a total of 31 *Synopeas* adults were reared, one from 2021 and 30 from 2023. The reared *Synopeas* spp. were binned into two morphotypes, one of which was confirmed to be *S. maximum* (n = 5). Of the five *S. maximum*, all of which were reared in 2023, one female was reared from the stems collected from the field near Syracuse, and the remaining adults (three females and one male) were reared from stems collected from the field near Wahoo. The other morphotype (n = 26), had not been observed in previous work in Minnesota (Melotto et al. 2023a, b). Of these 26 unidentified *Synopeas* sp., all of which were female, a single wasp was reared from stems collected in 2021, and 25 were reared from stems collected in 2023 (17 from the field near Syracuse and 8 from the field near Wahoo). For the single wasp reared in 2021, emergence timing was not recorded; however, for those reared in 2023, *Synopeas* adults emerged from 21 to 62 days after stems were collected. Finally, from those same emergence buckets, 792 and 1,989 adult *R. maxima* were reared in 2021 and 2023, respectively.

### DNA barcoding and wasp identification

**Figure 1.**
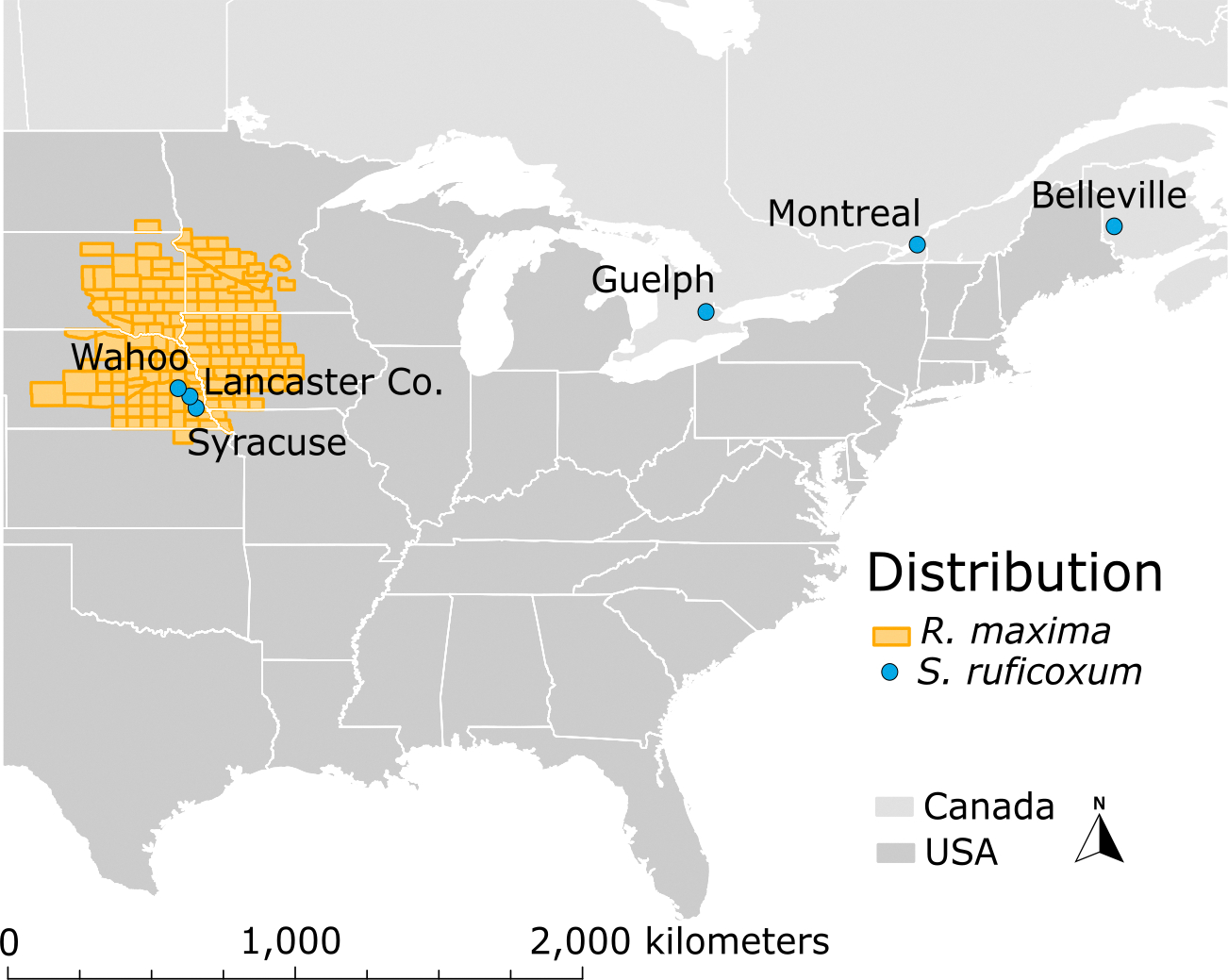
Geographic distribution of *Synopeas ruficoxum* and *Resseliella maxima* in the USA and Canada. Dots indicate locations where *S. ruficoxum* adults have been collected (Table 1). The holotype was collected in Belleville, Canada. Orange shading indicates the county-level geographic distribution of *R. maxima* (soybeangallmidge.org). Map was created in arcGIS Pro 3.3.0 using data obtained from the publicly available sources, including library.carleton.ca/find/gis/geospatial-data for state and province boundaries and soybeangallmidge.org for *R. maxima* distribution. All shapefiles were standardized to the WGS 1984 coordinate system.

Of the 26 unidentified *Synopeas* reared from stems collected from Nebraska, 23 were successfully barcoded using LCO/HCO primers (Table 1). Of the 23 COI sequences, 22 of them were 100% identical to one another (haplotype 1) and one was 99.5% identical to the rest (2 bp difference; haplotype 2) (Table 1). These two haplotypes were similar to two additional sequences on GenBank labeled as ‘Platygastridae sp.’ One of these Platygastridae sp. sequences was from a wasp collected in 2014 in the Montreal Botanical Garden, Montreal, Quebec, Canada (BOLD ID: POBGC998–15), and the other was collected in 2015 in the Arkell Research Station, Guelph, Ontario, Canada (BOLD ID: AGAKN602–17) (Fig. 1). These two additional sequences were identical to each other, 97.9% similar to haplotype 1, and 97.7% similar to haplotype 2, defining these two specimens as an additional haplotype (haplotype 3) of what appeared to be the same species.

The two Canadian specimens were borrowed, and all specimens of this unidentified *Synopeas* sp. (n = 28; two from Canada, 26 from Nebraska) were identified as *S. ruficoxum* by morphological comparison to the holotype (Fig. 1, Table 1). Since the original description of *S. ruficoxum* is based on a single female specimen and the description is brief (Buhl 2006), a redescription is provided below (see “Results: Taxonomy”). Finally, since only female *S. ruficoxum* have been collected, specimens were screened for *Wolbachia* using *Wolbachia*-specific primers. Out of the 23 wasps that were barcoded, 16 (70%) were PCR-positive for *Wolbachia* DNA, which does not fully align with the hypothesis that *Wolbachia* is mediating parthenogenesis (see Discussion).

### Phylogenetic analysis

**Figure 2.**
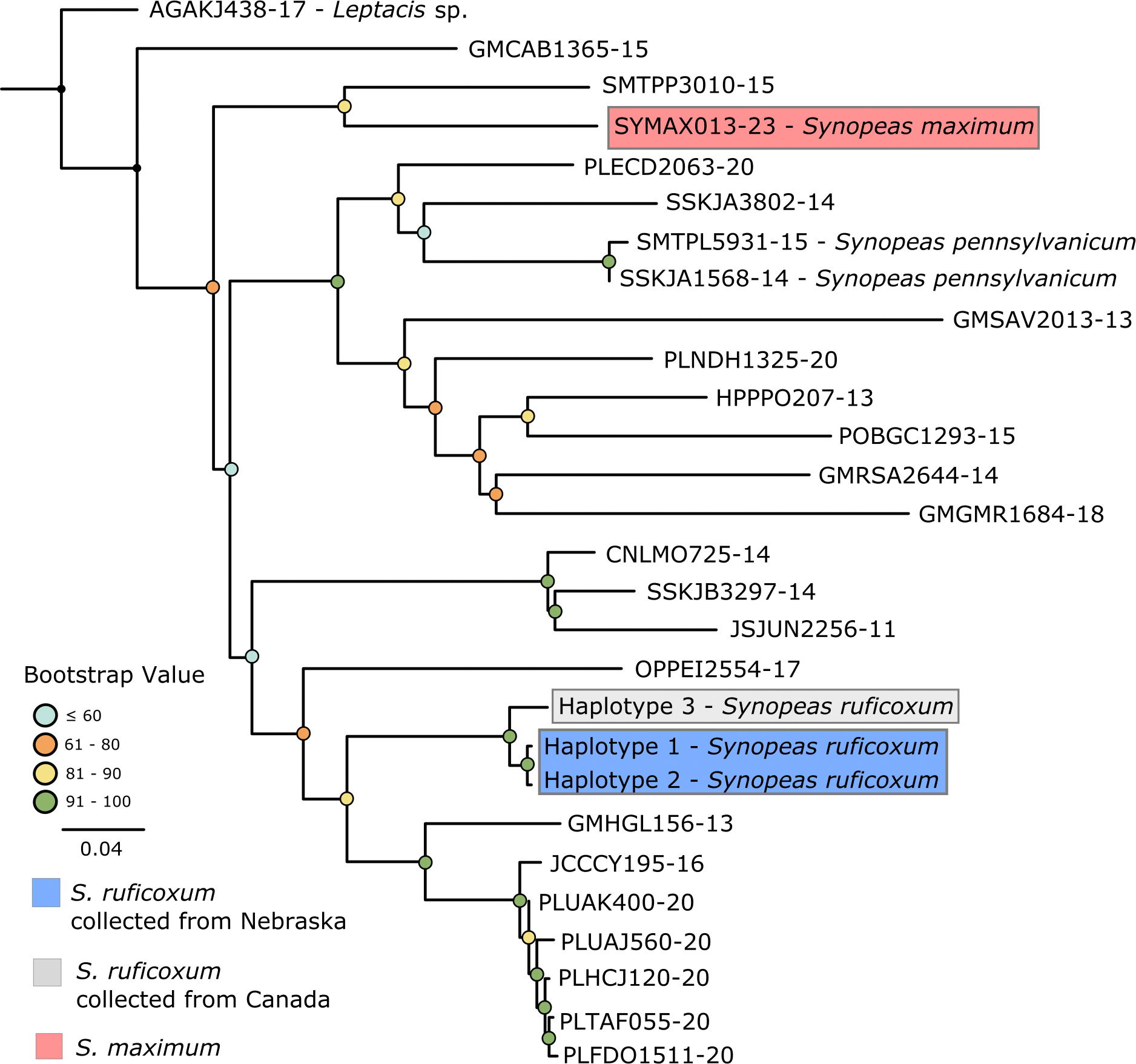
Phylogenetic tree of the genus *Synopeas* that focuses on *S. ruficoxum* collected from Nebraska and Canada, and *S. maximum*, another species associated with *R. maxima*. Collection localities associated with each sequence are available in Table 2. Nodes are color coded to indicate bootstrap support. Taxa are named by the barcode accession number (BOLD ID) and species name, if available. Branch lengths represent nucleotide substitutions per site.

A phylogeny of *Synopeas* was constructed from all unique *S. ruficoxum* haplotypes, *S. maximum*, and previously published *Synopeas* sequences (Fig. 2). While the backbone has relatively low bootstrap support (<60%), many species or putative species groups are strongly supported. Phylogenetic reconstruction supported *S. maximum* and *S. ruficoxum* as members of different species groups. This analysis supports the monophyly of *S. ruficoxum* and indicates that the Canadian haplotype is sister to the clade of USA haplotypes.

### Parasitoid-host association

Of seven field-collected *R. maxima* larvae, two screened positive for *S. ruficoxum* DNA. The *S. ruficoxum*-specific COI amplicons from these two specimens were sequenced, and both aligned with 100% identity to *S. ruficoxum* haplotype 1 (Table 1). The two larvae that were positive for *S. ruficoxum* were also barcoded, and were 99.5% similar to the corresponding COI region from the *R. maxima* mitochondrial genome (GenBank accession OQ342780) (Melotto et al. 2023c). Both *R. maxima* sequences were deposited in GenBank (accessions: PQ649846 and PQ649847). These results confirm the parasitoid-host association between *S. ruficoxum* and *R. maxima*.

### Taxonomy

**Figures 3.**
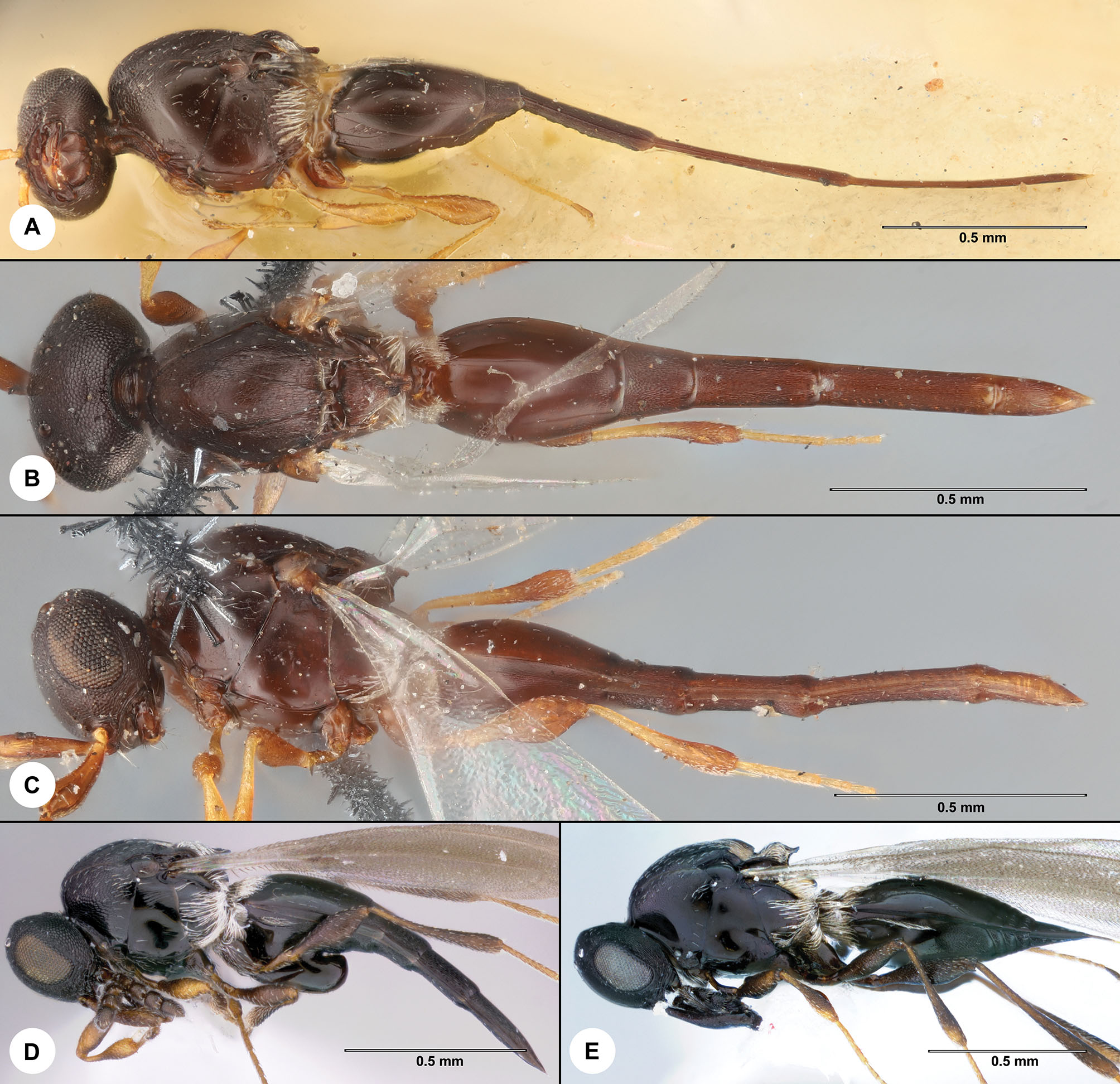
**A** *Synopeas idarniforme* (Dodd), holotype female, SAMA DB 32–032767, lateral view **B** *Synopeas craterum* (Walker) NHWM-HYM#0005311, dorsal view **C** *Synopeas craterum* (Walker) NHWM-HYM#0005311, lateral view **D** *Synopeas* sp. OSUC 404923 **E** *Synopeas* sp., OSUC 334240.

Elongation of the female metasoma, as seen in *S. ruficoxum*, was historically regarded as a genus-level character. *Dolichotrypes* Crawford & Bradley, 1911 was proposed for species with highly elongate and abruptly narrow T4–T6 (Fig. 3A); *Sactogaster* Förster, 1856 for species with S2 laterally compressed and ventrally expanded (Fig. 3D); and *Ectadius* Förster for species in which the metasoma is elongate, without modification to S2 or extreme elongation of T6 (Fig. 3B, C). In contrast, *Synopeas* sensu Förster has T2 longer than T3–T6 combined (Fig. 3E). *Synopeas ruficoxum* belongs to the group formerly treated as *Ectadius*, typified by *S. craterum* (Walker, 1835). We thus refer to the *craterum*-group for species in which female specimens have T5 longer than wide, T4–T6 not abruptly narrow, and S2 without a conspicuous ventral expansion.

We recognize 34 described species of *Synopeas* in the *craterum*-group (Table 3). The majority of these species were described from the tropics; only four are known from the Holarctic region: *S. craterum* from Europe; *S. abdominator* (Fouts, 1925) from the southern USA; *S. zaitama* Yoshida & Hirashima, 1979 from Japan; and *S. ruficoxum* Buhl, 2006 from Canada. Host associations are unknown for most species worldwide, except for *S. craterum*, which is associated with *Resseliella ribis* (Marikovskij, 1956) (Vlug 1995), and for *S. zaitama*, which is associated with *Resseliella odai* (Inouye 1955; Yoshida and Hirashima 1979). The present study adds a third host association for this group: *S. ruficoxum* and *R. maxima*.

The genus *Synopeas* is grammatically neuter, from the Greek σύν [syn], with, and ὄπεας [opeas], awl (Foerster 1856). The original epithet of *S. ruficoxus* is masculine, which is grammatically incorrect. This necessitates a mandatory change to the neuter form *S. ruficoxum*.

#### *Synopeas craterum* (Walker)

*Platygaster Craterus* Walker, 1835: 224 (original description).

*Ectadius craterus* (Walker, 1835) – Förster 1856: 113 (generic transfer).

*Polymecus craterus* (Walker, 1835) – Förster 1856: 144 (unnecessary replacement name); Marshall 1873: 19 (catalogued).

*Synopeas Craterus* (Walker): Thomson 1859: 71, 72 (generic transfer, description).

*Synopeas craterus* (Walker): Masner 1965: 141 (type information); Vlug and Graham 1984: 129 (lectotype designation); Vlug 1985: 205 (description of type, keyed); Vlug 1995: 77, 112 (catalogued, host information).

*Synopeas craterum* (Walker): Awad et al. 2023: 11, fig. 6 (mandatory change).

#### *Synopeas abdominator* (Fouts)

*Leptacis abdominator* Fouts, 1925: 101, 102 (original description).

*Synopeas abdominator* (Fouts): Masner and Muesebeck 1968: 98 (generic transfer, type information); Vlug 1995: 75 (catalogued).

#### *Synopeas zaitama* Yoshida & Hirashima

*Synopeas zaitama* Yoshida & Hirashima, 1979: 129–131, figs 43–49 (original description); Vlug 1995: 83 (catalogued).

#### *Synopeas ruficoxum* Buhl

Figs 4, 5

*Synopeas ruficoxa* Buhl, 2006: 203, figs 38–41 (original description).

*Synopeas ruficoxum* Buhl: von Gries et al. 2025 (mandatory change).

##### Description.

Females. Body length: 1.7–2.1 mm (n = 10). Body color: black. Color of legs: coxae brown, otherwise yellow to brown. Color of mesoscutellar spine: concolorous with mesoscutellar disc.

###### Head.

**Figure 4.**
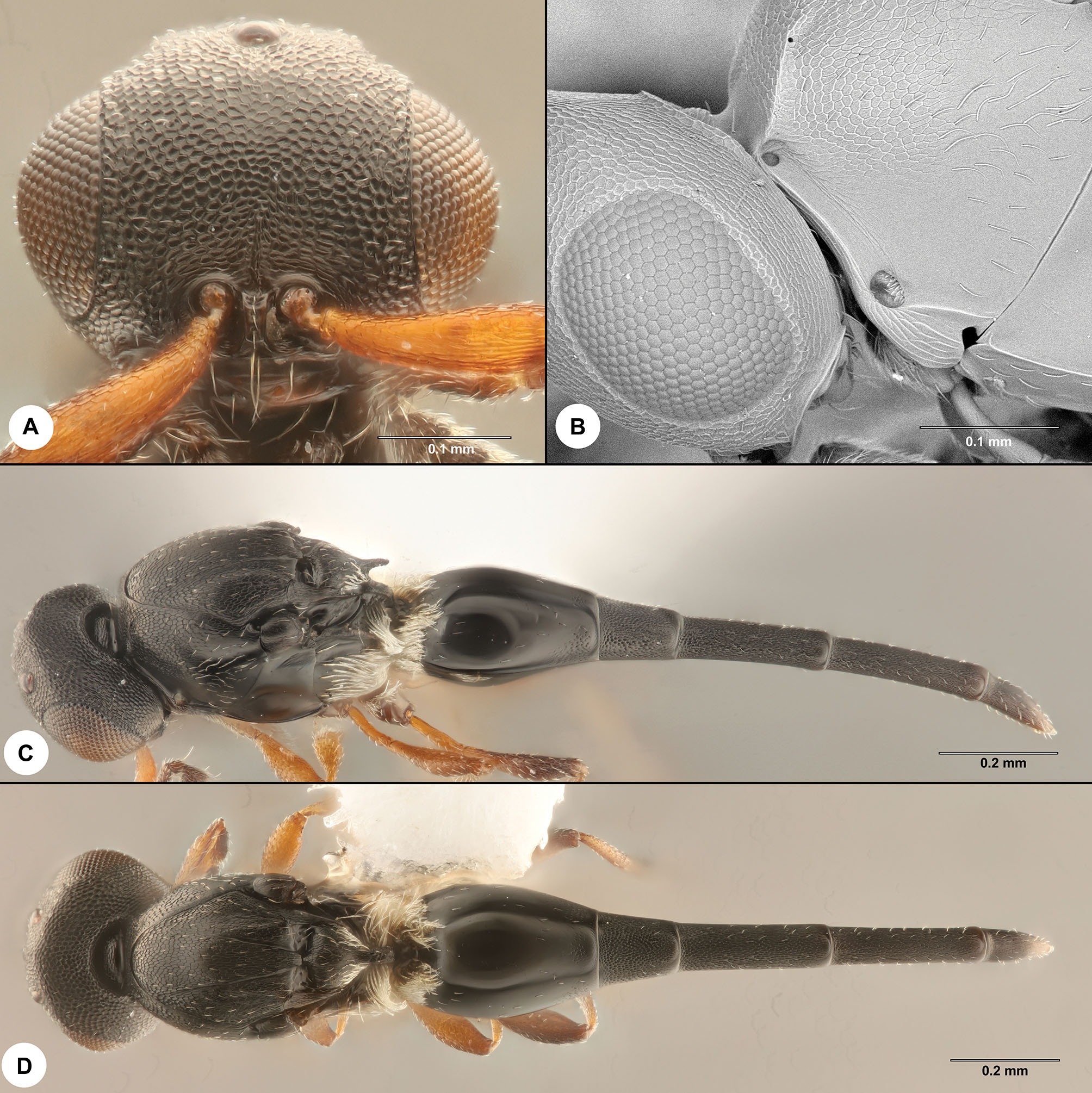
*Synopeas ruficoxum* (FSCA 00033423) **A** head, anterior view **B** head and pronotum, lateral view **C** habitus, dorsolateral view **D** habitus, dorsal view.

Shape of head in anterior view: round to ovoid (Fig. 4A). Central keel: absent; present only between toruli. Sculpture on frons: reticulate microsculpture. Epitorular sculpture: reticulate microsculpture; minute rugulae. Number of clypeal setae: 4. Length of median pair of clypeal setae: longer than lateral pair. Arrangement of clypeal setae: median pair closer to each other than to lateral setae. Shape of mandible: bidentate. Distance between lateral ocellus and compound eye (OOL): greater than 1 ocellar diameter. OOL: LOL: 1:1; 1:1.2. Lateral ocellar de-pression: present posterolaterally. Hyperoccipital carina: absent or only faintly sug-gested medially. Hyperoccipital carina strength: indicated as sharp angle of vertex between lateral ocelli. Distance between lateral ocellus and hyperoccipital carina: greater than 1 ocellar diameter. Claval formula: 1–1-1–1.

###### Mesosoma.

Epomial carina: present, complete, or nearly so. Pronotal cervical sulcus: smooth, glabrous. Anterior pronotal pit: present. Ventral pronotal pit: setose. Microsculpture of lateral pronotum: present anterodorsally, absent posteroventrally. Lateral pronotal sculpture coverage: 1/3–1/2. Setation of lateral pronotum: anteroventrally glabrous, otherwise uniformly sparse (Fig. 4B). Mesoscutellar spine: short to moderately developed and pointed. Mesoscutellar spine in lateral view: pointing posteriorly, often with slight downcurve at tip. Origin of mesoscutellar spine: slightly below dorsal apex of mesoscutellum. Posterior margin of propodeal carina in lateral view: rounded. Mesosomal dorsum in lateral view: slightly convex. Scutoscutellar sulcus: shallow, mesoscutum not elevated relative to mesoscutellum. Notauli: percurrent. Parapsidal line: indicated. Setation of mesoscutum: sparse. Mesoscutal lamella: short, truncate. Setation of mesoscutellum: sparse to absent, denser along posterior margin. Setal patch of dorsolateral hind coxa: present, long, extending dorsally to level of felt field.

###### Metasoma.

Sculpture of T2: faintly sculptured in posterior corners. Length of T2: conspicuously shorter than mesosoma. Sculpture of T3 to T5: reticulate. Sculpture of T6: entirely reticulate. Shape of T6: triangular, 2.5 times as long as wide. Microsculpture of S2: sculptured in posterior 1/3. Shape of S2: slightly expanded ventrally. Sculpture of S3 to S5: reticulate. Shape of S3: trapezoidal, approximately as wide as long. Shape of S4: more than twice as long as wide. Shape of S5: approximately twice as long as wide. Sculpture of S6: entirely reticulate.

###### Wing.

Length of setae on disc of fore wing: much shorter than distance between setal bases. Density of setae on disc of fore wing: sparse. Arrangement of setae on disc of fore wing: uniformly setose distally, proximally sparser. Fore wing marginal setae: uniformly very short.

##### Males.

Unknown.

##### Diagnosis.

*Synopeas ruficoxum* and *S. craterum* have distinctly elongate T4 and T5, both at least twice as long as wide (Figs 4C, D), as opposed to *S. abdominator* and *S. zaitama*, in which T4 is only slightly longer than wide. In *S. ruficoxum*, the mesoscutellar spine is well-developed, originating below the dorsal apex of the mesoscutellum, and points posteriorly, often with a downward curve at the tip. This sets it apart from *S. craterum*, which has a very short spine originating at the dorsal apex of the mesoscutellum, and from *S. abdominator*, in which the short, straight spine is angled posterodorsally. The posterior half of the lateral pronotum is smooth in *S. ruficoxum*, whereas it is sculptured in *S. zaitama*. The sculpture of the ventral metasoma is more extensive in *S. ruficoxum* than in *S. abdominator*, which has no sculpture on S6.

##### Remarks.

The original description compared *S. ruficoxum* to *S. auripes* (Ashmead, 1893) and *S. ashmeadii* Dalla Torre, 1898, neither of which shares its metasomal structure. Such comparisons are of little relevance and demonstrate the importance of examining specimens rather than relying solely on written descriptions. This is particularly relevant for very old descriptions because many authors provided too little detail for accurate diagnosis, and there may even be significant errors in the provided text and illustrations.

**Figures 5.**
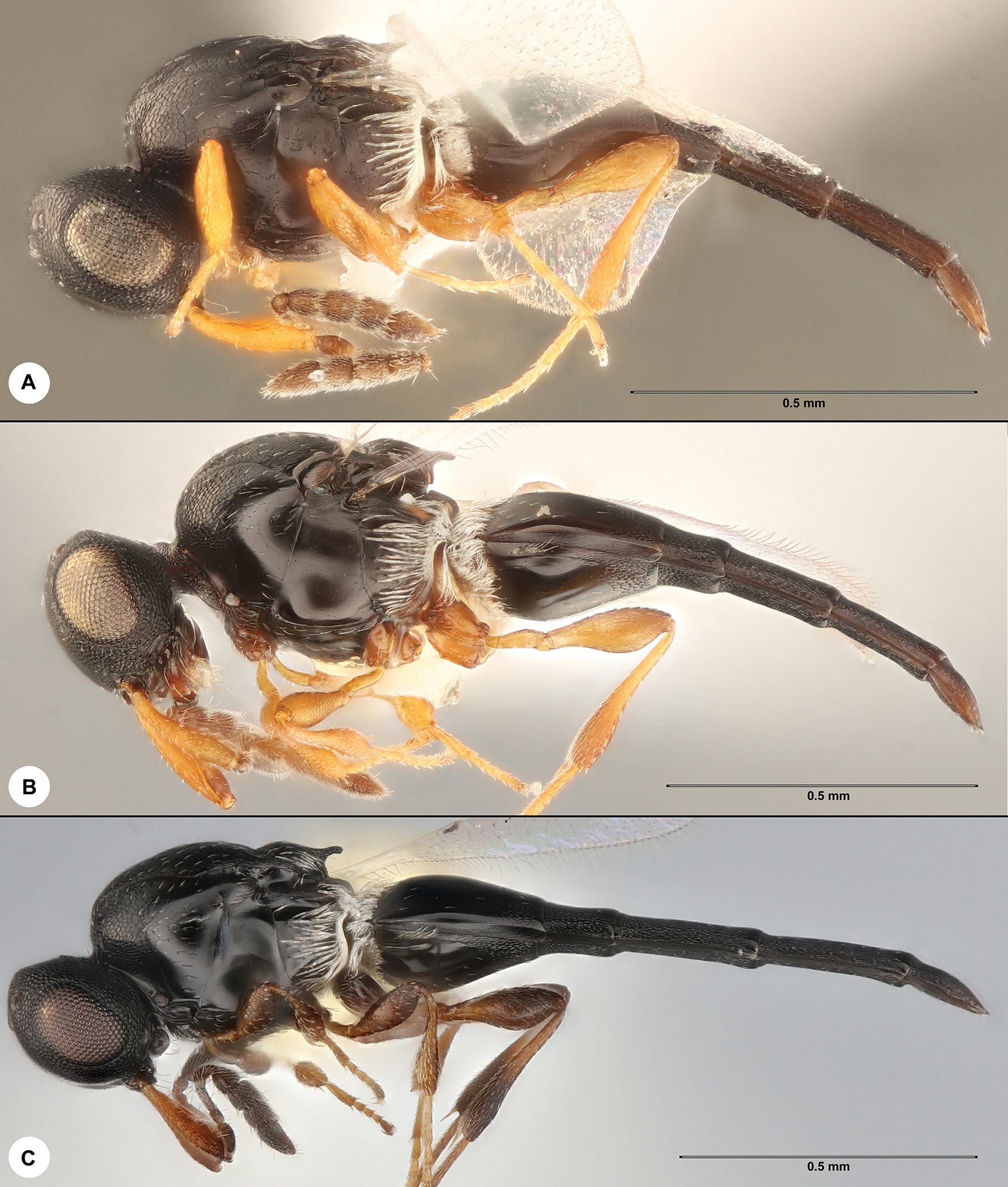
*Synopeas ruficoxum*, lateral view **A** holotype, New Brunswick, Canada (NHMD 918361) **B** Guelph, Ontario, Canada 2017 (BIOUG26568-F09; accession number MG346361) **C** Nebraska, USA 2021(FSCA 00034119).

The species epithet refers to the color of the coxae, which tend to be much lighter than the rest of the body (Fig. 5A, B). However, in some specimens the coxae are dark brown and the appendages are darker overall (Fig. 5C). Coloration can be altered by specimen age and preservation history, and also exhibits natural variation in many species. Due to this variability, coloration is not a reliable diagnostic character for most *Synopeas* species.

##### Material examined.

*Synopeas ruficoxum* Buhl, holotype female, NHMD 918361, Canada, New Brunswick, Carleton Co, Meduxnekeag River (near Belleville) 46.11354°N, 67.40556°W 10–15.VII.2005 Malaise trap 2 J. Bonet, M. Forshage, R. Hovmöller (ZMUC). Other material: 28 females, USA: Nebraska, FSCA 00033404–00033407 (CNCI); FSCA 00033408–00033411 (USNM); FSCA 00033412–00033419 (UMSP); FSCA 00033420–00033428, 00034119 (FSCA); BOLD Vouchers: BIOUG32277-G12, BIOUG26568-F09. The list of materials examined is also provided in Table 1.

## Discussion

Platygastrids have potential as biological control agents, but their implementation is impeded by the challenge of species-level identification and the lack of knowledge on their biology and how to rear them (Awad et al. 2025). The present study integrated morphology, molecular biology, and ecology to describe and provide identification resources for the platygastrid wasp, *S. ruficoxum*. Furthermore, our integrated approach enabled us to determine that *S. ruficoxum* is the second-known parasitoid of *R. maxima*. While parasitism of *R. maxima* by *S. ruficoxum* was suggested by its emergence from buckets containing field-collected soybean stems infested with *R. maxima*, DNA barcoding was critical for providing direct evidence of the host-parasitoid association. This is only the third host association known for the *Synopeas craterum*-group, all of which parasitize *Resseliella* species (Yoshida and Hirashima 1979; Vlug 1995). However, to further evaluate the biological control potential of *Synopeas* species, assessments of (a) host specialization, (b) reproductive strategy, and (c) evolutionary and life history are needed.

Since there are limited known host associations for *Synopeas* generally, there is a poor understanding of the relative degree of host specialization. Because there are challenges associated with such assessments (i.e., rearing of multiple species of plants, cecidomyiids, and parasitoids) (Awad et al. 2025), host associations might best be explored by implementing molecular methods. However, an exclusively molecular biology approach is limited primarily by the ability to associate COI sequences with taxon names. Combining paired collections of cecidomyiids and parasitoids (e.g. rearing insects from plant material) with a much-needed revision of platygastrid taxa would enhance the utility of this approach. Furthermore, in addition to enabling the identification of host associations, an integrated approach (including molecular biology, ecology, and systematics) would also allow us to determine the parasitoid community of specific cecidomyiid species.

However, confidently identifying all associated parasitoids could still prove difficult, as individual cecidomyiid species have up to 14 parasitoid species associated with them (Hawkins and Gagné 1989). Across *Resseliella* species (n = 8), a maximum of two associated parasitoids have been recorded (Hawkins and Gagné 1989). Our current understanding of *R. maxima* is in line with this range, as two species of *Synopeas* have been confirmed to parasitize *R. maxima*: *S. maximum* (Melotto 2023a, b), and now *S. ruficoxum*. However, parasitism has only been assessed in two out of the seven states with known *R. maxima* infestations. Notably, *S. ruficoxum* has been reared from *R. maxima* only in Nebraska, whereas *S. maximum* has been collected from both Nebraska and Minnesota. Despite only being reared from Nebraska, other records of *S. ruficoxum* extend from Ontario to the Atlantic coast of Canada, indicating that *S. ruficoxum* may attack other species of cecidomyiids, or, that *R. maxima* may have a geographic range larger than presently documented. Indeed, low-level, asymptomatic infestations of *R. maxima* can go unnoticed (Bragard et al. 2023).

Not only are there potential differences in host specialization and geography, sex-ratios indicate the two *Synopeas* species have different reproductive biologies, an important factor that impacts biological control programs (Stouthamer 1993; Heimpel and Mills 2017). Only females parasitize hosts, so a sexual population with males means an appreciable proportion of individuals will not contribute directly to pest suppression (Ode and Hardy 2008). Additionally, in the context of biological control agent releases, males facilitate mating with wild populations and ultimately the potential to dilute desirable traits originally present in the released population (Hopper et al. 1993). This challenge is further intensified by the potential for rapid post-release evolution, which may lead to shifts in agent efficacy or host specificity (Roderick and Navajas 2003). While both male and female *S. maximum* have been collected, all records of *S. ruficoxum* are female. The *S. maximum* adults collected from the field in Wahoo consisted of three females and one male, which is more female-biased than what was observed in Luverne, Minnesota (seven females, nine males) (Melotto et al. 2023a). However, these are small sample sizes that may not necessarily reflect the population-level sex ratio. Regardless, the consistent presence of *S. maximum* males indicates that this species is sexually reproducing. In contrast, the fact that only female *S. ruficoxum* have been collected suggests this is a species that reproduces via thelytokous parthenogenesis (i.e., the asexual reproduction of females). We screened *S. ruficoxum* for *Wolbachia*, to assess whether the all-female population was potentially due to *Wolbachia*-mediated parthenogenesis (Fricke and Lindsey 2024b). However, *Wolbachia* was detected in only 70% of *S. ruficoxum*, which does not fully support the hypothesis that *Wolbachia* is inducing parthenogenesis. It is possible *Wolbachia* prevalence was disrupted by high summer temperatures, as has been seen in other species (Pintureau et al. 2002), or asexual reproduction is simply not mediated by *Wolbachia* (Fricke and Lindsey 2024a). In either case, determining how the reproductive biologies of *S. maximum* and *S. ruficoxum* impact parasitism rates and population dynamics requires further research.

Thelytoky also has taxonomic implications (Enghoff 1976; Stouthamer et al. 1990). The closest relative of a parthenogenetic species may be one that reproduces sexually, making it crucial that female-to-female comparisons are made when constructing diagnostic tools. Furthermore, some parasitoids are known to be geographically parthenogenetic (Brues 1928). This phenomenon is not yet known in Platygastridae, but it also has yet to be explored. Some Nearctic *Synopeas* species were described solely on male specimens, precluding the use of important features of the female antenna and metasoma for identification. Thus, species names from male-only descriptions can be a taxonomic hindrance and, in some cases, may be treated as *nomina dubia*. As described here, the *craterum*-group of *Synopeas* is defined by metasomal characters found only in female specimens. The reliability of metasomal shape for subgeneric classification, either as formal subgenera or informal species groups, has yet to be evaluated in a phylogenetic context. Such an endeavor requires a much larger taxon sampling than provided here and multiple loci, but likely will be very useful for parsing *Synopeas* into smaller groups that are monophyletic and more manageable from an identification standpoint.

Independent of their use for classification, modifications to the metasoma in *Synopeas* offer the opportunity to explore functional morphology. Presumably, the shapes of the metasoma and ovipositor reflect parasitoid oviposition strategy, with metasomal elongation providing extended reach of the ovipositor. The two parasitoids of *R. maxima* may have specialized on different developmental stages of the host. For example, *S. maximum*, with a short metasoma (Melotto et al. 2023a), could attack hosts earlier in development and closer to the surface of the plant tissues, whereas *S. ruficoxum*, with a long metasoma, may target hosts at a later stage of development, when the larvae have burrowed deeper into the plant tissue (Gagné et al. 2019). The distance between *S. maximum* and *S. ruficoxum* in the phylogenetic analysis (Fig. 2) suggests that host shifts within *Synopeas* may be determined by ecological and physical factors more than phylogenetic affinity. However, further observational studies are needed from a greater representation of parasitoids and hosts.

## Conclusions

In summary, this work identified *S. ruficoxum* as the second parasitoid of *R. maxima*, which provides a host-parasitoid system in which we can explore interesting ecological questions. One key question is whether the two *Synopeas* parasitoids differ in phenology, such as generation cycles or population density, and how this may influence their effectiveness in controlling *R. maxima*. Additionally, determining spatial variation in the abundance of the two *Synopeas* parasitoids and *R*. *maxima* could inform our understanding of shifts in host parasitoid communities across geographic regions. Finally, investigating interactions between *S. ruficoxum* and *S. maximum*, including niche partitioning and multiparasitism, could reveal how they coexist and jointly impact host populations.

## Figures and Tables

**Table 1.** Specimens of *Synopeas ruficoxum* examined.

Lab Code	Collecting Unit Identifier	Collection Location	Year Collected	Haplotype†	BOLD ID

NA	NHMD 918361 (holotype)	Belleville, Canada	2005	NA	NA
GMP#04688	BIOUG26568-F09	Montreal, Canada	2014	3	POBGC998-15
GMP#07677	BIOUG32277-G12	Guelph, Canada	2015	3	AGAKN602-17
PN12	FSCA 00034119	Lancaster Co., NE	2021	NA	NA
WB27	CNC664038	Syracuse, NE	2023	1	SRSVG001-24
WB28	CNC664039	Syracuse, NE	2023	1	SRSVG002-24
WB29	CNC664040	Wahoo, NE	2023	1	SRSVG003-24
WB30	CNC664041	Syracuse, NE	2023	1	SRSVG004-24
WB31	USNMENT01977476	Syracuse, NE	2023	1	SRSVG005-24
WB32	USNMENT01977477	Syracuse, NE	2023	1	SRSVG006-24
WB33	USNMENT01977478	Syracuse, NE	2023	1	SRSVG007-24
WB34	USNMENT01977479	Syracuse, NE	2023	1	SRSVG008-24
WB35	FSCA 00033412	Syracuse, NE	2023	1	SRSVG009-24
WB36	FSCA 00033413	Wahoo, NE	2023	1	SRSVG010-24
WB38	FSCA 00033428	Wahoo, NE	2023	NA	NA
WB40	FSCA 00033414	Wahoo, NE	2023	1	SRSVG011-24
WB41	FSCA 00033415	Wahoo, NE	2023	1	SRSVG012-24
WB43	FSCA 00033416	Syracuse, NE	2023	1	SRSVG013-24
WB44	FSCA 00033417	Syracuse, NE	2023	1	SRSVG014-24
WB45	FSCA 00033418	Syracuse, NE	2023	1	SRSVG015-24
WB46	FSCA 00033419	Syracuse, NE	2023	1	SRSVG016-24
WB47	FSCA 00033420	Syracuse, NE	2023	1	SRSVG017-24
WB48	FSCA 00033421	Syracuse, NE	2023	1	SRSVG018-24
WB49	FSCA 00033422	Syracuse, NE	2023	1	SRSVG019-24
WB50	FSCA 00033423	Wahoo, NE	2023	1	SRSVG020-24
WB51	FSCA 00033424	Wahoo, NE	2023	1	SRSVG021-24
WB52	FSCA 00033425	Syracuse, NE	2023	1	SRSVG022-24
WB55	FSCA 00033426	Wahoo, NE	2023	2	SRSVG023-24
WB56	FSCA 00033427	Syracuse, NE	2023	NA	NA

† Used in phylogenetic tree. NA indicates specimens without sequence data.

**Table 2.** Seqeunces used in phylogenetic reconstruction.

BOLD ID	BOLD Taxonomy†	BOLD Collection Localities

AGAKJ438-17	*Leptacis* species	Canada, Ontario, Guelph
GMCAB1365-15	Platygastridae	Costa Rica, Guanacaste, Area de Conservacion Guanacaste
SMTPP3010-15	*Synopeas* species	Canada, British Columbia, Fort St. James
PLECD2063-20	Platygastridae	Costa Rica, Guanacaste, Area de Conservacion Guanacaste
SSKJA3802-14	*Synopeas* species	Canada, Nova Scotia, Kejimkujik National Park
SMTPL5931-15	*Synopeas pennsylvanicum*	Canada, Manitoba, Winnipeg
SSKJA1568-14	*Synopeas pennsylvanicum*	Canada, Nova Scotia, Kejimkujik National Park
GMSAV2013-13	Platygastridae	South Africa, Gauteng
PLNDH1325-20	Platygastridae	Costa Rica, Guanacaste, Area de Conservacion Guanacaste
HPPPO207-13	*Synopeas* species	Canada, Nova Scotia, Halifax
POBGC1293-15	*Synopeas* species	Canada, Quebec, Montreal
GMRSA2644-14	Platygastridae	Russia, Primorskiy Kray
GMGMR1684-18	*Synopeas* species	Germany, Bavaria, Munich
CNLMO725-14	*Synopeas* species	Canada, Quebec, La Mauricie National Park
SSKJB3297-14	*Synopeas* species	Canada, Nova Scotia, Kejimkujik National Park
JSJUN2256-11	Platygastridae	Canada, Ontario, Leeds and Grenville
OPPEI2554-17	Platygastrinae	Canada, Ontario, Owen Sound
GMHGL156-13	Platygastrinae	Honduras, Cortes, Cusuco National Park
JCCCY195-16	Platygastrinae	Costa Rica, Guanacaste, Area de Conservacion Guanacaste
PLUAK400-20	Platygastrinae	Costa Rica, Guanacaste, Area de Conservacion Guanacaste
PLUAJ560-20	Platygastrinae	Costa Rica, Guanacaste, Area de Conservacion Guanacaste
PLHCJ120-20	Platygastrinae	Costa Rica, Guanacaste, Area de Conservacion Guanacaste
PLTAF055-20	Platygastrinae	Costa Rica, Guanacaste, Area de Conservacion Guanacaste
PLFDO1511-20	Platygastrinae	Costa Rica, Guanacaste, Area de Conservacion Guanacaste

† Listed here at the lowest taxonomic rank provided on BOLD.

**Table 3.** World species of the Synopeas craterum-group (Ectadius sensu Förster).

Species	Year	Type repository	Type Locality	Images

*S. abdominator* (Fouts)	1925	USNM	USA: Texas	USNMENT00954758
*S. atturense* Mukerjee	1981	USNM	India	USNMENT01109823
*S. bengalense* Mukerjee	1978	USNM	India	USNMENT01109922
*S. bennetti* Buhl	2011	NHMUK	Trinidad	
*S. craterum* (Walker)	1835	NMINH	England	https://zenodo.org/records/13931982
*S. fontali* Buhl	2002	MNCN	Panama	
*S. grenadense* (Ashmead)	1895	NHMUK	Grenada	https://zenodo.org/records/13935191
*S. guatemalae* Buhl	2003	MZLU	Guatemala	
*S. halmaherense* Buhl	2008	NBC	Indonesia	https://zenodo.org/records/4503235
*S. indopeninsulare* Mani	1975	USNM	India	USNMENT01109919
*S. infuscatum* Buhl	2008	NBC	Indonesia	https://zenodo.org/records/4503181
*S. insulare* (Ashmead)	1894	NHMUK	St. Vincent	
*S. longifuniculus* Buhl	2002	MNCN	Panama	
*S. macrurus* (Ashmead)	1895	NHMUK	Grenada	https://zenodo.org/records/13935199
*S. masneri* Buhl, O’Connor & Ashe	2009	NMINH	Indonesia	https://zenodo.org/records/4563030
*S. mineoi* Buhl, O’Connor & Ashe,	2009	NMINH	Indonesia	https://zenodo.org/records/4539460
*S. mukerjeei* Buhl	1997	NHMD	Philippines	https://zenodo.org/records/4503979
*S. nievesaldreyi* Buhl	2002	MNCN	Panama	
*S. nigricorne* Buhl	2015a	NHMD	Chile	https://zenodo.org/records/14194534
*S. nigroides* Buhl	2001	MZLU	Ecuador	
*S. orbitaliforme* Buhl	2011	NHMUK	Trinidad	
*S. polaszeki* Buhl	2004a	NHMD	Cote d’Ivoire	https://zenodo.org/records/14201398
*S. politiventre* Buhl	2015a	NHMD	Chile	https://zenodo.org/records/1037312
*S. popovicii* Buhl	2015b	NHMD	Madagascar	https://zenodo.org/records/14199492
*S. rionegroense* Buhl	2004b	HNHM	Argentina	
*S. royi* Buhl	2001	MZLU	South Africa	
*S. ruficoxum* Buhl	2006	NHMD	Canada	https://zenodo.org/records/14037325
*S. saintexuperyi* Buhl	1997	NHMD	Papua New Guinea	https://zenodo.org/records/4501968
*S. saltaense* Buhl	2009	HNHM	Argentina	
*S. saopaulense* Buhl	2004b	HNHM	Brazil	
*S. solomonensis* Buhl	1997	NHMD	Solomon Islands	
*S. striatum* (Risbec)	1958	RMCA	DRC	
*S. tanzanianum* Buhl	2010	NHMD	Tanzania	
*S. zaitama* Yoshida & Hirashima	1979	KUEC	Japan	https://zenodo.org/records/14193063
